# Active women over 50: study protocol for RCT of a low-dose information and support program to promote physical activity behaviour change

**DOI:** 10.1186/s12889-019-7514-6

**Published:** 2019-09-04

**Authors:** Geraldine Wallbank, Catherine Sherrington, Colleen G. Canning, Leanne Hassett, Roberta Shepherd, Bethan Richards, Catherine Mackay, Anne Tiedemann

**Affiliations:** 1 Institute for Musculoskeletal Health, School of Public Health, Faculty of Medicine and Health, The University of Sydney, PO Box M179 Missenden Road, Camperdown, Sydney, 2050 Australia; 2 Discipline of Physiotherapy, Faculty of Health Sciences, The University of Sydney, PO Box 170, Lidcombe, Sydney, NSW 1825 Australia; 3 Institute of Rheumatology and Orthopedics, Royal Prince Alfred Hospital, Level 4 Royal Prince Alfred Hospital, Camperdown, Sydney, 2050 Australia; 4 Workplace Health and Safety, Royal Prince Alfred Hospital, Level 7 KGV Building, Missenden Rd, Camperdown, Sydney, 2050 Australia

**Keywords:** Exercise, Behaviour change, Health, eHealth, Workplace, Physical activity

## Abstract

**Background:**

There is compelling evidence that physical activity has many physical and mental health benefits and can delay the development of disability in older age. However, uptake of this health behaviour is sub-optimal in working women in their middle age. This trial aims to establish the impact of a low-dose information program, incorporating follow-up support using behaviour change techniques, compared with a wait-list control group, on physical activity among women aged 50+ years.

**Methods:**

100 female university or health service employees aged 50 years and over who are not sufficiently active according to national guidelines will be recruited and randomised to: [1] attend one information session at the worksite with follow-up email support and provision of resources including use of an activity tracker (*Fitbit*) for 3 months and free trial class at the university sports facility, or [2] a wait-list control to receive the intervention after the 3-month follow-up period. The primary outcome will be the proportion of people achieving 10,000 steps/day at 3 months post randomisation. Secondary outcomes will include the proportion of people achieving national guideline-recommended physical activity levels, the average self-reported hours of physical activity per week, perceived benefits of and barriers to exercise participation, physical functioning, and mood. Analyses will be planned, conducted while masked to group allocation and will use an intention-to-treat approach.

**Discussion:**

This randomised controlled trial will evaluate the impact of a simple intervention using behaviour change techniques to increase physical activity participation in insufficiently active working women over the age of 50.

**Trial registration:**

ACTRN12617000485336, prospectively registered, approved 04/04/2017.

## Background

Physical inactivity is an important, but modifiable public health problem that can substantially impact health and independence [1, 2]. Globally 5.3 million deaths per year are attributable to inactivity [3], a public health issue that is growing in size [4] and that has an estimated economic cost to the health-care system of INT$67.5 billion worldwide [5]. Physical inactivity in older age requires urgent attention, with 80% of Australian women over the age of 75 years insufficiently active [6], and the proportion of older people in the population rapidly rising. By 2050 up to 25% of the Australian population (10.5 million people) will be aged 65 years and over [1, 7].

There is compelling evidence that physical activity has many physical and mental health benefits [8–10]. Physical activity has been described as “the best buy in public health” [7] with “large unused potential … in the global prevention of non-communicable diseases, including dementia” [11]. National physical activity guidelines provide clear guidance on the amount of physical activity required to maximise health and wellbeing [8]. Australian adults are recommended to accumulate 150 to 300 min of moderate intensity or 75 to 150 min of vigorous intensity physical activity, or an equivalent combination of both moderate and vigorous activities each week, and to do muscle strengthening activities on at least 2 days each week [8].

Health conditions that could be ameliorated with physical activity are particularly common in older people. There is emerging evidence that regular physical activity participation delays the development of disability in older women by up to 15 years [9] but the uptake of this health behaviour remains sub-optimal, particularly among working women compared to their male counterparts [12]. Women are more likely than men to have high daily sitting time [13] which puts them at additional risk for chronic ill health. Almost half of the female workforce are over the age of 50 years [14], and while many become more active with retirement, this may be too late to prevent disability in older age. Commencement of appropriate physical activity by women in their 50s should therefore be a priority and workplaces are an ideal target.

Women over 50 years have unique barriers to becoming more active. They may have changing carer responsibilities from their older and more independent children to their older but more dependent parents, may face the demands of work in senior roles or re-entering the workforce, may be managing chronic health conditions, or may never have incorporated sufficient physical activity in their lives before [14]. Hence, this population requires a targeted and supported approach to ensure behaviour change is sustainable [15].

Work accounts for around 60% of an adults’ waking hours [16] and is a contributor to the risk of physical inactivity [17], an additional problem for women who are already more sedentary than their male counterparts. Healthy workplaces that target employee wellbeing, that is, employee physical, emotional, mental and social health, are positively associated with increased productivity, performance, reduced absenteeism, and health care utilisation [18]. The benefits of participating in physical activity addresses employee wellbeing [17], and so makes the workplace an ideal setting to provide an intervention to increase physical activity participation in this target group.

People who are insufficiently physically active can be supported to adopt and sustain physical activity with the implementation of a behavioural change framework. Michie, van Stralen and West [19] developed the Behaviour Change Wheel framework (BCW) and COM-B model of behaviour change; with capability, opportunity and motivation as key factors that interact to influence behaviour. Physical and psychological capability, the physical and social opportunities that lie outside the individual, and a person’s habits, emotions and decisions which influence their motivation, all interplay to affect whether a person engages in a behaviour. The BCW framework can be used as a tool to guide the design of interventions aimed at changing behaviour.

Education is an intervention function within the BCW. It can be delivered in different forms (e.g. workshops, printed brochures, the internet) to increase health literacy and support behavioural change. Education can provide a person with the psychological capability to change their behaviour and provide the motivation through addressing their individual beliefs about capabilities and consequences [19]. In the primary care setting, education with brief advice and motivational interviewing increased self-reports of physical activity but practitioners reported it was time consuming to deliver [20]. Providing physical and social opportunities to change behaviour through workplace-based interventions can increase physical activity, but the optimal type of intervention and delivery format is yet to be established [21].

Activity trackers are small, commercially available and relatively inexpensive wearable devices that clip onto clothing or are worn around the wrist and have grown in popularity. They have been shown to be an effective strategy to increase physical activity participation in the general population, particularly when there has been a daily step goal of 10,000 steps [22].

We have designed a scalable, simple low-dose intervention, utilising the BCW, to enhance physical activity participation in women aged 50 years and older. The aim of this randomised controlled trial is to test the impact of the intervention on physical activity participation in working women.

## Methods

**Fig. 1:**
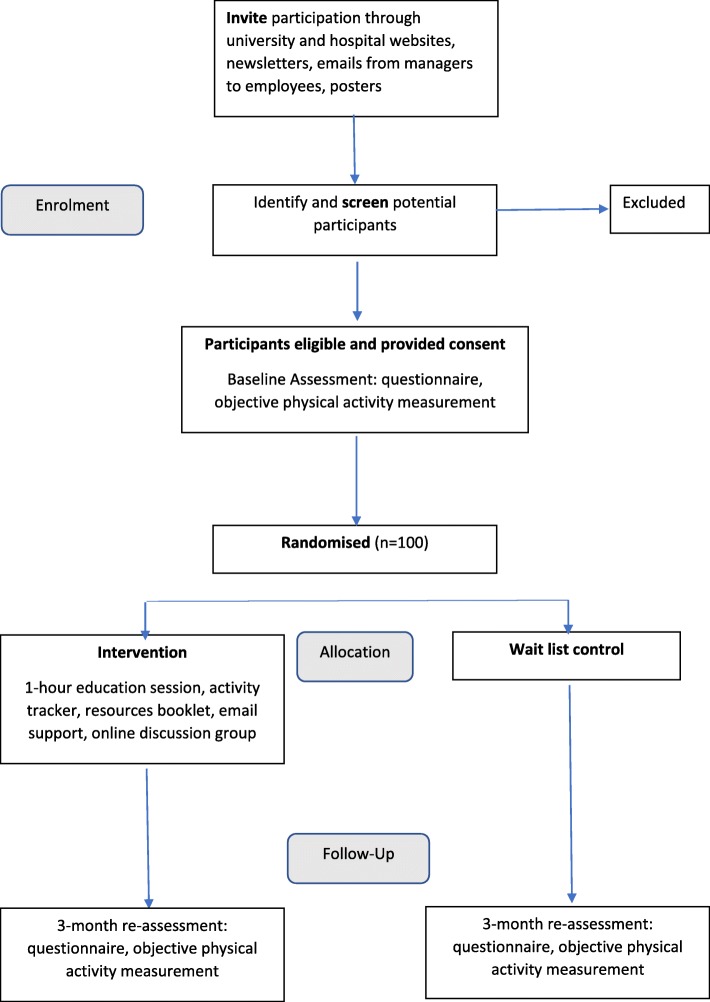
Trial design

We will conduct a randomised controlled trial with wait-list control. The design of the trial is illustrated in Fig. 1. This trial has been designed according to the Consolidated Standards Of Reporting Trials (CONSORT) statement [23], and is reported according to the Standard Protocol Items: Recommendations for Interventional Trials (SPIRIT) statement [24], and with reference to the Template for Intervention Description and Replication (TIDieR) checklist [25].

One hundred women will be randomised to either receive the intervention immediately, or after the 3-month follow-up period. People will be eligible for inclusion in the trial if they are female, aged 50 years and over, employees of The University of Sydney or Sydney Local Health District in Australia. Potential participants will be excluded if they have limited English language skills, or have a medical condition that precludes participation in regular physical activity, or are already sufficiently active in accordance with the Australian Physical Activity guidelines [8]. Potential participants will be provided with a participant information statement outlining the study procedures. All participants will sign a consent form before participating in the study.

Participant recruitment will be through advertising using the workplace channels at the university and health service. This will be via university newsletters and websites, the health service intranet, posters and email invitations sent to managers of university and hospital departments for distribution to their employees. No workplace incentives will be offered for participation in this study. People who wish to participate in the research project will be screened for eligibility either over the telephone or via email.

Participants will be enrolled into the study after they have completed the initial screening, provided informed written consent to participate in the project, and have completed all baseline questionnaires and measurements of physical activity.

Baseline questionnaires will be sent to participants via an online survey link (*REDCap*) or by post, as preferred by the participant. Baseline physical activity will be measured with an *Actigraph* accelerometer. A research team member who is blind to study group allocation will download the accelerometer data. Each participant will receive a study enrolment number upon completing the baseline questionnaire generated by the *REDCap* database and this will be recorded on all study documents.

Following completion of the baseline measures, participants will be randomly allocated to:

- participation in one information session plus follow-up support immediately (intervention group) or
- participation in one information session plus follow-up support after the 3-month follow-up period (control group)

A variable block randomisation schedule will be prepared from a computer-generated list of random numbers by a researcher not involved in recruitment. The randomisation schedule will incorporate stratification for recruitment site (University campus, Sydney Local Health District) or pairs. Where there are two participants who are known to each other or if they are from the same workplace department as revealed by the initial contact, they will be randomised to the same group to reduce cross-contamination of the results. The randomisation schedule will be embedded in a secure online database (*REDCap*) to achieve allocation concealment.

The intervention group will attend a one-hour information session that will provide: 1) information about the importance of physical activity for maximising physical and mental health and preventing disability; 2) group discussion in overcoming the barriers to physical activity; 3) information and handbook of resources about existing opportunities for women over 50 years to participate in; 4) video case-study interviews of four female staff members aged over 50, and one female staff member aged over 80 who have overcome barriers to increase their physical activity; 5) tools to enhance physical activity participation (e.g. physical activity recording charts, guidance on goal setting), 6) the use of a pedometer or internet-connected activity tracker (*Fitbit*) for the duration of the study, to provide motivation and feedback to increase activity levels if desired, and 7) a free trial class at the university sports facility. An online discussion group will be set up for participants who wish to remain in contact, and a fortnightly email from the research team will be sent to provide information, motivation and support. The information session and follow-up email support will emphasise strategies to overcome barriers to participation. These will include incorporating physical activity into daily life through active transport, incidental physical activity and benefits to health and habit formation of even small increases in physical activity. The information session materials and sample physical activity recording charts will also be made freely available to participants.

The handbook of resources includes a list of local physical activity opportunities including on-campus activities and facilities for university and health service staff; websites for physical activity information and opportunities for the over 50s including a telephone-based coaching service; local council services and facilities; and free health and fitness applications available for smartphone devices. Information session participants will receive a pre-exercise screening tool and exercise intensity guidelines produced by *Exercise Sports Science Australia*, *Fitness Australia*, and *Sports Medicine Australia* [26] and will be advised to seek individual advice from a health professional if they have any concerns about increasing their physical activity or if they have an injury.

Participants will attend the information session during their lunch hour at their workplace. For participants who are not able to attend in person, a video conferencing facility will be provided. For participants unable to attend the scheduled session times, a recording of the session will be provided via an online webinar link. These participants will be able to email or phone the presenters with any questions after viewing the webinar. The information session will be presented by physical activity researchers and sports facility staff who will also facilitate discussion. Table 1 summarises the content of the intervention using the TIDieR checklist [25].

**Table 1:** Intervention description using the Template for Intervention Description and Replication (TIDieR) checklist [25]

1. Brief name	Active Women over 50 physical activity education and promotion intervention
2. Why	Uptake of physical activity is sub-optimal in working women in their middle age. Women over 50 years have unique barriers to becoming more active (eg. higher sedentary time, greater carer responsibilities, work demands).
3. What materials	The education program has been designed for women over the age of 50 using the Behaviour Change Wheel framework and COM-B system model of behaviour change [20] to facilitate and sustain behavioural change. Information will be presented via a Powerpoint slideshow outlining the evidence on benefits of physical activity, overcoming barriers to physical activity, physical activity options available to university and health service staff, getting started and maintaining activity. Video case-study interviews of four female staff members aged over 50 years who have overcome barriers to increase their physical activity will be shown to inspire participants. Participants will receive a printed handbook containing physical activity resources such as internet-based information and programs, local physical activity programs and facilities, suggestions of the numerous free applications for smartphone devices, and a guide to starting physical activity. Participants will also receive a pre-exercise screening tool and exercise intensity guidelines [28]; will be offered the use of a pedometer or internet-connected activity tracker (*Fitbit*) for the duration of the study, to provide motivation and feedback to increase activity levels; will have access to 1) an online discussion group with other participants to share ideas and for motivation, 2) fortnightly emails reinforcing the workshop content and supporting participants to increase their physical activity, 3) a free trial class at the Sydney University Sports Facility, and 4) all the information session materials (slides, video case study interviews, University sports facility brochures) via an email link. The internet-based information resources will include the *Get Healthy* website produced by the NSW Ministry of Health [29] (www.gethealthynsw.com.au), *Make Healthy Normal* website produced by the NSW Ministry of Health (www.makehealthynormal.com.au); internet-based program resources may include the *Active and Healthy* website [30], *parkrun* website promoting free, weekly community walk/runs [31].
4. What procedures	An information session will be used to provide education and support to participants to increase their physical activity through university and health service on-campus and local opportunities. Participants will have the option to loan a *Fitbit* activity tracker for the duration of the study, and/or receive follow up fortnightly emails, and/or access to an online discussion group *Yammer.* All participants will be emailed a link to access the presentation materials, and will have email access to the research team for any further enquiries.
5. Who provided	Research team members with expertise in the field of physical activity research and practice will provide and deliver the information session and facilitate discussion. A University sports facility staff member will outline their available programs at the information session.
6. How	Information sessions will be face-to-face in a group setting of up to 20 people at a time. A video conferencing facility will be offered to participants who are not able to physically attend the session location but will allow them to be able to interact with the group. Information sessions will also be offered via a pre-recorded internet link for participants who are not able to attend the sessions in person, and will have the opportunity to ask any questions to the research team via email or telephone.
7. Where	The information sessions will be held at the workplace at two university campus sites and at one hospital site in Sydney, in a meeting room with data projection and video-conferencing facilities. Remote access will be through a video conferencing facility or through a pre-recorded online link of the webinar.
8. When and how much	The intervention will be one, 1-h information session scheduled during lunch time (12-1 pm). Those attending via the online link will attend at a time of their convenience. Follow up support emails will be sent fortnightly to participants who elect to receive these. There will be no cost.
9. Tailoring	All participants will receive the same information session content and access to the same internet-based resources. Participants will have the opportunity to ask questions during the session or via email or telephone support following it. Participants will be advised to seek individual advice from a health professional if they are concerned about commencing physical activity or have an injury.

Participants randomised to the control group will be allocated to a waiting list and will receive access to the intervention after the 3-month follow-up period.

The primary outcome will be the proportion of people achieving an average of at least 10,000 daily steps, measured objectively with a matchbox-sized waist-worn accelerometer (*Actigraph GT3X+*) at 3-months. *ActiGraph GT3X+* is able to accurately estimate how physically active a person is throughout the day by measuring 3D body accelerations and is a valid instrument that has been extensively researched in the physical activity and public health field [27]. Participants will be instructed to wear the accelerometer on the right hip, attached via an adjustable elastic belt, for seven consecutive days during waking hours (except during water-based activities or bathing), and to complete an activity log. Activity counts per second will be collected at a sampling frequency of 30 Hz and reintegrated to 60s epochs for data analysis.

The primary outcome will be quantified as the average step counts per day. This outcome will detect any increase in incidental physical activity (i.e. less time spent sitting) as well as adoption of more formal exercise regimens. These data will be collected over a 7-day period to account for day-to-day variation in physical activity levels. The accelerometers will be posted to participants with clear instructions for use and email or telephone support will be available. Participants will be provided with pre-paid envelopes to return the devices to the research centre. Accelerometer data will be manually checked against participant activity logs to verify wear time, and erroneous data will be excluded prior to analysis. Physical activity participation will be assessed at baseline and 3 months after participant randomisation, and *ActiGraph* data will be extracted by a research assistant who is unaware of group assignment (i.e. blinded outcome assessment).

Accelerometer data will be analysed using *ActiLife 6* software. Acceptable wear time will be defined as 4 days or more of 10 h or more per day. Periods of 90 min or more of consecutive zeros (indicating non-use) will be considered as non-wear time.

The secondary outcomes will be assessed at baseline and at 3 months post-randomisation via a self-report questionnaire that will take approximately 30 min to complete. The secondary outcomes will include: 1) the proportion of people achieving adequate physical activity levels as recommended by national physical activity guidelines: 150 to 300 min of moderate intensity physical activity or 75 to 150 min of vigorous intensity physical activity, or an equivalent combination of both moderate and vigorous activities, each week, and to do muscle strengthening activities on at least 2 days each week [7], measured using an *Actigraph* accelerometer; 2) the average total number of hours of physical activity per week, self-reported with the International *Physical Activity Questionnaire* (IPAQ) [32]; 3) change in perceived benefits of and barriers to exercise participation, as measured by the *Exercise Benefits and Barriers Scale* [33]; 4) physical functioning as measured by the function component of the *Late Life Function and Disability Instrument* (LLFDI) [34]; 5) mood as measured with the *Positive and Negative Affect Schedule* (PANAS) [35]. Participants in the intervention group will also complete a follow-up questionnaire evaluating whether the suggested or other resources were investigated or participated in, whether goals were set, and participants’ perceived barriers and plans following the workshop.

Odds ratios will be calculated to assess the effect of group allocation on the dichotomously-scored primary outcome (proportion of people achieving 10,000 steps/ day) and secondary outcome (proportion of people achieving physical activity in accordance with national guidelines). General linear models will assess the effect of group allocation on the continuously-scored secondary outcomes (self-reported physical activity, perceived exercise benefits and barriers, physical functioning and mood), adjusting for baseline scores.

A *p*-value of < 0.05 will be considered statistically significant. A full statistical analysis plan will be devised by the lead investigator prior to commencement of data analysis. Analyses will be pre-planned, conducted while masked to group allocation and will use an intention-to-treat approach. Analyses will be conducted using the *Stata 14* software package.

A total of 50 participants per group (i.e. 100 participants) will provide 80% power to detect 30% more people in the intervention group reaching the recommended 10,000 steps/ day than the control group. This calculation assumed a proportion of 27% compliance with the 10,000 steps/ day activity level in the control group, dropout rate of 15% and alpha of 5%. The estimates of the proportion of people achieving 10,000 steps/ day activity level was taken from the baseline results of the University of Sydney staff participating in the Global Corporate Challenge [36]. This sample size is also expected to be sufficient to detect between-group differences in the order of 10–15% for the secondary outcome measures.

Participant data will be collected by survey questionnaires, completed online or in paper format if preferred, and via *Actigraph* activity monitor posted to participants at baseline and 3-month follow-up. An email or phone call will be sent to any participants with incomplete outcome measures to improve participant retention. To ensure participant confidentiality, the final dataset will contain re-identifiable information only. Demographic information linking the participant to the data will be stored on a separate file. All data will be entered onto a password protected database and maintained on a firewall protected local network server at The University of Sydney. Paper files will be stored in a locked filing cabinet in the Chief Investigators office. Access to all data will be limited to authorised study staff. All publications associated with the results of the study will involve de-identified data, so participant confidentiality will be maintained.

The trial protocol has been approved by the Human Research Ethics Committee at The University of Sydney, Australia (approval number 2017/115) and Research Ethics and Governance Office at Sydney Local Health District (Protocol number X17–0316; LNR/17/RPAH/473, LNRSSA/17/RPAH/560, LNRSSA/18/RPAH/46, LNRSSA/18/RPAH/75). The trial is registered (ACTRN12617000485336). Results will be disseminated via peer-reviewed journal articles and international conferences, and a lay summary will be made available to all participants at the completion of the study.

## Discussion

The evidence for the physical and mental benefits of physical activity, even in small amounts [9, 37, 28] in delaying the development of disability and chronic disease is compelling, yet the problem of physical activity participation remains a significant global public health dilemma.

This study targets women over the age of 50, a group that is at greatest risk of developing disability in older age, who are insufficiently active according to the national physical activity guidelines [8]. The trial will provide evidence on the impact of a brief, low-dose, low-cost intervention to address the problem of physical inactivity in the workplace setting, an ideal setting due to the amount of time spent at work, the potential risk that work may contribute to inactivity, and possible benefits that the intervention may have on employee wellbeing [17].

The intervention will provide support for behaviour change through the BCW framework. Behavioural change and adherence to the program will be facilitated, enabled and supported by providing the group information session face-to-face or online, opportunities for discussion, inspiration through video case studies, post-workshop support with the provision of a resource handbook, use of an activity tracker (*Fitbit*) and workshop materials, receiving fortnightly email messages, joining an online discussion group, and a free trial class at the university sports facility.

A limitation of this trial is that participants will be recruited based on self-rated physical activity prior to objective physical activity baseline measures. Other limitations are that only land-based physical activities will be measured by the accelerometer, a lack of individual tailoring of the intervention, and limited generalisability of this sample to other workplace settings and to men.

However, if found effective, this low-cost program with supported follow-up, could be scalable, directly implemented into the workplace setting and directly address the problem of physical inactivity. There would also be scope for this intervention to be translated to the broader community by exploring other settings and resource delivery.

An effective and scalable preventive health strategy is needed to ease the burden on health systems already at capacity, and burden on individuals’ lives. This simple intervention has the potential to address the problem of physical inactivity to delay the onset of disability in older age.

## Data Availability

Not applicable.

## Abbreviations

BCW: Behaviour Change Wheel
CONSORT: Consolidated Standards Of Reporting Trials statement
SPIRIT: Standard Protocol Items: Recommendations for Interventional Trials statement
TIDieR: Template for Intervention Description and Replication checklist
